# Automated Oxygen Delivery in Neonatal Intensive Care

**DOI:** 10.3389/fped.2022.915312

**Published:** 2022-06-22

**Authors:** Vrinda Nair, Prakash Loganathan, Mithilesh Kumar Lal, Thomas Bachman

**Affiliations:** ^1^Neonatal Intensive Care Unit, South Tees Hospitals National Health Service (NHS) Foundation Trust, James Cook University Hospital, Middlesbrough, United Kingdom; ^2^Translational and Clinical Research Institute, Faculty of Medical Sciences, Newcastle University, Newcastle upon Tyne, United Kingdom; ^3^School of Biomedical Engineering, Czech Technical University in Prague, Prague, Czechia

**Keywords:** automated oxygen, hyperoxemia, hypoxemia, oxygen saturation, preterm

## Abstract

Oxygen is the most common drug used in the neonatal intensive care. It has a narrow therapeutic range in preterm infants. Too high (hyperoxemia) or low oxygen (hypoxemia) is associated with adverse neonatal outcomes. It is not only prudent to maintain oxygen saturations in the target range, but also to avoid extremes of oxygen saturations. In routine practice when done manually by the staff, it is challenging to maintain oxygen saturations within the target range. Automatic control of oxygen delivery is now feasible and has shown to improve the time spent with in the target range of oxygen saturations. In addition, it also helps to avoid extremes of oxygen saturation. However, there are no studies that evaluated the clinical outcomes with automatic control of oxygen delivery. In this narrative review article, we aim to present the current evidence on automatic oxygen control and the future directions.

## Introduction

Oxygen is a drug with a narrow therapeutic range in vulnerable preterm neonates. Avoiding both hypoxemia and hyperoxemia is important especially in neonates as both are associated with short-term and long-term adverse outcomes (1, 2). Hypoxemia causes cellular damage, and this may be associated with poor outcomes such as death or disability (3–5). Hyperoxemia causes oxygen toxicity and oxidative stress that has been implicated in the development of bronchopulmonary dysplasia (BPD) and retinopathy of prematurity (ROP) (6–8). Although, there are recommendations for the oxygen saturation (SpO2) targeting in preterm infant, it is challenging in the routine practice to keep the SpO2 in the prescribed target range (TR) (9, 10). Traditionally the Fractional inspired oxygen (FiO2) is often needed to be titrated by the bedside staff (Manual control, M-FiO2) to try to maintain the SpO2 in the TR. The compliance with manual control is hugely variable across centers (11). Studies have shown that M-FiO2 results in a considerable proportion of time spent outside of the TR of SpO2. The preterm infants in view of their cardiorespiratory instabilities and apnea of prematurity, are prone to fluctuations in SpO2 and intermittent episodes of hypoxemia (12). In a prospective study, reporting achieved vs. intended SpO2 targets in preterm infants <28 weeks, only 16–64% of time infants were in the intended range and above the range 20–73% of time (11). With the advent of automated system in oxygen delivery (A-FiO2), the challenges to maintain the SpO2 in the TR has been overcome to an extent. A-FiO2 uses real time continuous SpO2 data to makes necessary adjustments in FiO2 based on algorithms that differ with devices and systems.

Studies using A-FiO2 have consistently shown to improve the proportion of time spent in the TR of SpO2, reduce hypoxemia and hyperoxemia in preterm infants on non-invasive or invasive respiratory support. Whilst the A-FiO2 systems have been commercially available, it has not yet established itself in the routine care in the neonatal ICU (2, 13). This is indicative of the challenges with its use, and more importantly the lack of clinical outcome data with the use of A-FiO2.

In this review article we will make a case for importance of SpO2 targeting in preterm infants, clinical implications of intermittent hypoxemia/hyperoxemia, current evidence for the use of A-FiO2, the types of algorithms available in clinical practice, challenges in implementation of technology and the future directions.

## SpO2 Targeting in Preterm Infants

In the last decade, five large randomized controlled trials (14–18) were conducted to evaluate the optimal SpO2 TR in preterm infants. Following this, three systematic reviews (19–21) including Cochrane review (9) and one individual patient meta-analysis (10) have been published. There was no difference in the primary composite outcome of death or major disability at 18–24 months corrected age between the lower SpO2 group (85–89%) and higher SpO2 group (91–95%). The lower SpO2 group was associated with higher risk of mortality and NEC. The risk of ROP was higher in the higher SpO2 group. However, there was no difference in the rates of severe visual impairment (22). Interestingly, the separation of SpO2 between the two groups in these studies was less than expected with significant overlap in SpO2 in the two groups. The current recommendations by international bodies suggest the use of 90–95% as SpO2 TR in preterm infants until 36 weeks Post menstrual age (20, 23).

## Effects of Hypoxemia and Hyperoxemia

In *post-hoc* analysis of Canadian oxygen trial (COT study), intermittent prolonged hypoxemia (SpO2 < 80%) for at least 1 min was associated with increase in composite outcome of death after 36 weeks or major neuro-disability (RR 1.66, 95% CI: 1.35–2.05) at 18 months corrected age (5). Jensen et al. in their *post-hoc* analysis also showed increased risk of severe BPD with both the frequency of severe hypoxemic episodes and duration of hypoxemia (4). Compared with infants with lowest decile of hypoxemic episodes, infants with highest number of hypoxemic episodes (10th decile) had an adjusted relative risk of 20.40 (95% CI: 12.88–32.32) for severe BPD.

Oxygen supplementation and hyperoxemia, whilst on supplemental oxygen, has been associated with ROP, BPD and PVL (24, 25). Hyperoxemia, mostly an overshoot to the oxygen supplementation following a hypoxemic event, is a preventable by strict adherence to the SpO2 target or by the use of A-FiO2.

Whilst it is important to maintain the SpO2 in TR for preterm infants, it is equally imperative to avoid hypoxemia and hyperoxemia. Hence it is essential to choose and adhere to the appropriate alarm limits for the SpO2 TR (26). A-FiO2 studies have shown an advantage of A-FiO2 over M-FiO2in reducing extremes of SpO2.

## Algorithms for A-FiO2

The A-FiO2 works on the principles of continuous SpO2 monitoring using pulse oximeter, regular feedback into the rule-based algorithms and changes in FiO2 delivery based on this feedback. The algorithms vary in designs and hence the frequency and magnitude of changes to FiO2 is variable across the various A-FiO2 devices. The designs include on adaptive model control algorithms, proportional integral differential algorithm and state machine control algorithm (27).

The state machine control algorithm is based on a set of rules. The algorithm uses the difference between the desired and the actual SpO2, its velocity and acceleration as input. The incorporated rules then set out a FiO2 change by the controller. In the proportional integral differential algorithms, the controller calculates the difference between the desired and the actual SpO2 (error), integrates over time and velocity and determines the oxygen output. The adaptive model algorithms consider the infant's physiology that may have the effect on oxygen dissociation curve. A non-linear model is created based on FiO2-oxygen saturation relationship. The controller adjusts its model of this relationship to achieve target saturations (28).

**Figure 1:**
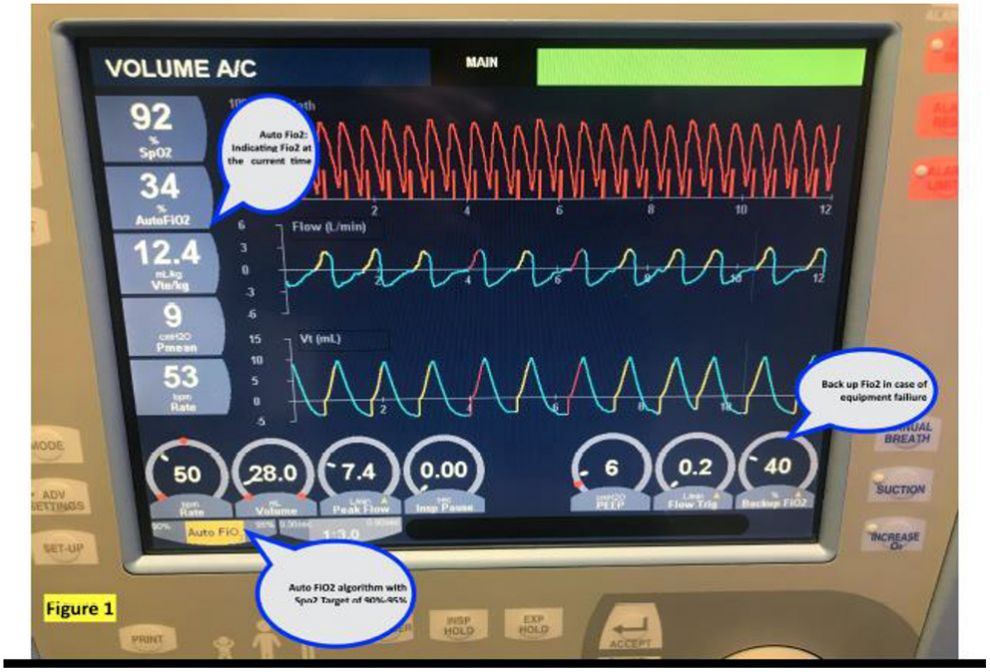
${\text{CLiO}}_{2}^{™}$ integral to the Avea^Ⓡ^ infant ventilator.

**Figure 2:**
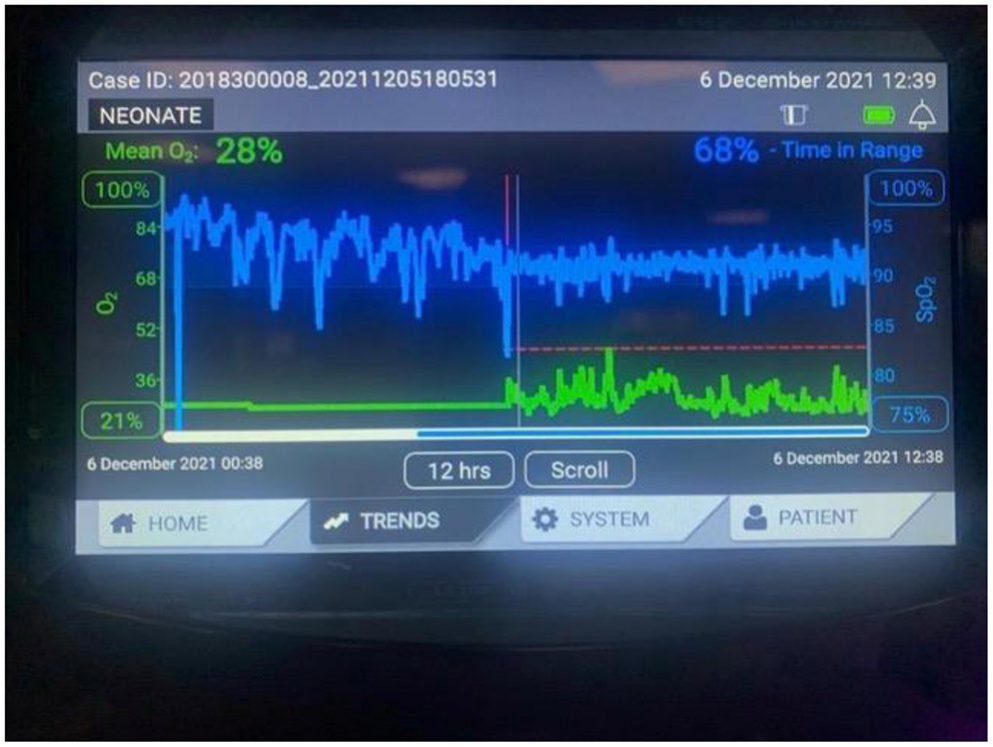
Oxygen assist module in Vapotherm Precision Flow Device.

The currently available algorithms include ${\text{CLiO}}_{2}^{™}$ integral to the Avea^Ⓡ^ infant ventilator (Figure 1), CLAC (Closed Loop automatic oxygen control) incorporated into the Leoni ventilator, IntellO2™ in the Oxygen assist module in Vapotherm Precision Flow (Figure 2), OxyGenie on SLE6000 ventilator, PRICO on the Fabian acutronic ventilators and SPOC on Sophie neonatal ventilator (MEDACX).

## Current Available Evidence for the Use of A-FiO2 in Neonates

The details of the currently available studies are shown in Table 1 (29–45). Majority of these studies were cross-over RCT. The SpO2 targets used were variable across the studies, as were the post-natal age at entry and the algorithms used. All the studies were of short duration varying from 2 to 48 h. Six of these studies included infants on invasive ventilation, another six used a combination of invasive ventilation and nasal continuous positive airway pressure (NCPAP), and further six studies only included infants on non-invasive respiratory support (NCPAP or High Flow therapy). The primary outcome in most was the proportion of time in SpO2 TR. The studies consistently reported significantly higher proportion of time in SpO2 TR, lower proportion of time below & above the SpO2 TR and reduced need for manual adjustments with A-FiO2. In a recent systematic review with 13 studies, A-FiO2 resulted in increased time spent in target SpO2 of 85–96% [MD = 8.96; 95% CI (6.26, 11.67), *p* < .00001], and 90–95% [MD = 18.25; 95% CI (4.58, 31.65), *p* = 0.008] (46). A-FiO2 reduced the time in hypoxemia [SpO2 < 85%; MD = −1.24; 95% CI (−2.05, −0.43), *p* = 0.003] and hyperoxemia [SpO2 > 98%; MD = −0.99; 95% CI (−1.74, −0.25), *p* = 0.009].

**Table 1:** Characteristics of A-FiO2 studies.

**References**	**Study design**	**Study population**	**Primary outcome (automatic vs. manual)**	**Other outcomes (automatic vs. manual)**
Claure et al. (29)	Randomized cross over trial for 2 h on each mode.	*N* = 14 Mechanically ventilated Very Low Birth weight infants	Increase in time spent in TR	No significant difference in other outcomes.
Claure et al. (30)	Randomized cross over trial for two 4-h periods	*N* = 16 Mechanically ventilated preterm infants and receiving supplemental oxygen	Increase in time spent in TR	Decrease in time above the TR Decrease in time SpO2 ≥ 98% Decreased time with SpO2 <88%
Claure et al. (31)	Randomized cross over trial for 2 consecutive 24-h periods	*N* = 32 Mechanically ventilated preterm infants and receiving supplemental oxygen	Increase in proportion of time spent in TR	Decrease in time spent in SpO2 > 98% Decrease in time SpO2 <87% Decrease in number of FiO2 changes No difference in time spent in SpO2 <80% or <75%
Lal et al. (32)	Randomized cross over trial for 2 consecutive 12-h periods	*N* = 27 Mechanically ventilated Preterm infants <32 weeks on supplemental oxygen	Increase in proportion of time spent in TR	Decrease in proportion of time in SpO2 below the TR Decrease in proportion of time in SpO2 above the TR Decrease in proportion of time in SpO2 <80 Decrease in proportion of time in SpO2 ≥ 98
Morozoff et al. (33)	Cross over study with three algorithms with manual control	*N* = 7 Mechanically ventilated preterm infants	Increase in proportion of time spent in TR	Decrease in number of hypoxemic episodes Decrease in number of manual adjustments.
Sturrock et al. (34)	Randomized cross over trial for 2 consecutive 12-h periods	*N* = 24 Mechanically ventilated preterm infants at a corrected gestation age <6 months	Decrease in number of desaturations with SpO2 <85% lasting >30 and >60 s	Increase in proportion of time spent in TR. Decrease in proportion of time in SpO2 below the TR Decrease in proportion of time in SpO2 above the TR
Hallenberger et al. (35)	Randomized cross over trial for 24-h period.	*N* = 34 Preterm infants either mechanically ventilated or on NCPAP and receiving supplemental oxygen.	Increase in proportion of time spent in TR	Decrease in proportion of time below the TR No difference in time above the TR Decrease in number of manual FiO2 adjustments
van Kaam et al. (36)	Randomized cross over trial for 24 h each and randomized to two SpO2 targets	*N* = 80 Preterm infant <33 weeks on invasive or non-invasive respiratory support	Increase in proportion of time spent in TR	Decrease in proportion of time spent below TR and SpO2 <80% Decrease in number of episodes with SpO2 <80% for >1 min
Waitz et al. (37)	Randomized cross over trial for 24 h each	*N* = 15 Preterm ventilated infants	Increase in proportion of time spent in TR	Decrease in number of prolonged (>60 sec) episodes with SpO2 <88% Decrease in proportion of time spent in SpO2 > 96%
Gajdos et al. (38)	Randomized cross over trial for 12 h period.	*N* = 12 Very Low Birth weight infants	Increase in proportion of time spent in TR	Decrease in time spent below the TR Decrease in number of episodes in SpO2 <88% for >180 s No difference in time spent above TR, median FiO2 and tissue oxygenation.
Schwarz et al. (39)	Randomized cross over trial: Three modes: CLAC_fast_, manual control only, manual control with CLAC_slow_	*N* = 19 Preterm infants <34+1-week gestation receiving respiratory support (invasive or non-invasive) and supplemental oxygen	Increase in time spent in TR (CLAC fast vs. manual)	Decrease in time spent below the TR (CLAC fast vs. manual)
Urschitz et al. (40)	Randomized cross over trial of 90 min for three group. - Routine manual control. - Optimal manual control. - FiO2 Controller	*N* = 12 Preterm infants on NCPAP and receiving supplemental oxygen	Increase in time spent in TR with A-FiO2 as compared to routine M-FiO2	Decrease in manual adjustments of FiO2 with A-FiO2
Plottier et al. (41)	Non-randomized study with 4-h intervention with A-FiO2 with	*N* = 20 Preterm infants on non-invasive	Increase in proportion of time spent in TR	Decrease in time spent below the TR, above the TR, SpO2 <80%. SpO2 > 98%
	total of 8 h manual control (4 h before and after automated oxygen).	support and supplemental oxygen		Decrease in number of changes to oxygen therapy
Dargaville et al. (42)	Cross over study with 24-h intervention with automated oxygen with total of 24 hrs manual control (12 h before and after automated oxygen)	*N* = 35 Preterm infants on non-invasive respiratory support and supplemental oxygen	Increase in proportion of time spent in TR	Decrease in time in SpO2 <80%. Decrease in time spent in severe and prolonged hyperoxemia and hyperoxemia
Zapata et al. (43)	Randomized trial with total study duration of 12 h	*N* = 20 Preterm infants <30 weeks and <1,000 grams receiving supplemental oxygen with nasal cannula	Increase in time spent in TR	Decrease in time spent in SpO2 > 95% Reduced need for manual adjustments
Reynolds et al. (44)	Randomized cross over trial	*N* = 30 Preterm infants on High Flow Nasal Cannula with FiO2 ≥25%.	Increase in time spent in TR	Decreased number of prolonged episodes of SpO2 <80% No difference in number of episodes/hours of SpO2 >98%
Dijkamn et al. (45)	Randomized cross over trial for 2 consecutive 24-h periods	*N* = 27 Preterm infants <30 weeks on High Flow Nasal Cannula and FiO2 >0.25	Increase in proportion of time spent in TR	Decrease in proportion of time spent below TR, above TR and SpO2 No difference in time spent in SpO2 > 98%

Various algorithms are available with A-FiO2. Only two studies compared different A-FiO2 algorithms. Schwarz et al. compared fast and slow CLAC algorithms (39) and Salverda et al. compared OxyGenie controller (SLE6000 ventilator) with CLiO2 controller (AVEA ventilator) in randomized cross over trial (47). In the latter study 15 preterm infants received each intervention for 24 h in a cross over fashion. Time spent in the SpO2 TR were higher with OxyGenie with median time of 80.2 (72.6–82.4) % vs. 68.5% (56.7–79.3%) in CLiO2 algorithm. With OxyGenie time spent above the TR were lower (6.3 vs. 15.9%, *p* < 0.005) and time spent below the TR (14.7 vs. 9.3%, *p* < 0.05) were higher as compared to CLiO2. The difference in the hypoxemia and hyperoxemia episodes may be related to the different design of the algorithm.

Although A-FiO2 has consistently shown to be superior to the M-FiO2 in maintaining the SpO2 in the TR, we do not know if this physiological benefit is associated with improved clinical benefits. It can be hypothesized that better control in maintaining SpO2 in TR, reduction in hypoxemia and hyperoxemia may concomitantly result in improved short- and long-term clinical outcomes. There are currently no studies available that has looked at use of A-FiO2 to improve clinical outcomes. For a clinical outcome study with A-FiO2, it is imperative that parallel arm RCT design is chosen. The study should also capture the entire period on respiratory support and supplemental oxygen. A large RCT with aim to recruit 2,340 preterm infants (<28 weeks) is currently underway (NCT03168516) (48). In this clinical outcome study, infants are randomized to either A-FiO2 or M-FiO2, continue to be in randomized arm as much as time possible without any crossover. Primary outcome of this RCT is composite outcome of death or severe ROP, BPD or NEC. This study has another primary outcome of composite of death or any of the following: language or cognitive delay, motor impairment, severe visual impairment or hearing impairment all assessed at 2 years of age.

An improvement in saturation targeting with A-FiO2 was not associated with improved tissue oxygenation in studies by Dani et al. and Waitz et al. (37, 49).

Alarms are necessary evils in any intensive care units (50). Alarm overloads can result in fatigue and desensitization among staff which in turn could pose a clinical risk. Studies with A-FiO2 have shown a significant lower alarm rate as compared to M-FiO2 (32). The frequency of alarms in A-FiO2 can be further reduced with much looser alarm limits (51). The reduction in alarm frequency may help in reducing the nursing workload and possibly increase cognitive attention. However, it is imperative to consider the appropriate alarm threshold for SpO2 and FiO2 so as to alert the caregivers of a deterioration.

Few centers have implemented A-FiO2 for routine care of preterm infants. Van Zanten et al. reported outcomes of before and after implementation of A-FiO2 (52). Although there was a significant improvement in time spent in the SpO2 TR, there was no difference in duration of respiratory support and mortality. Salverda et al. also reported pre (2012–2015; *N* = 293) and post (2015–2018; *N* = 295) implementation of A-FiO2 in preterm infants (53). There was no difference in any of the clinical outcomes like ROP, NEC, BPD, and duration of hospital stay. Both these studies by the nature of their design were not powered for these outcomes.

Van Zanten et al. also reported that the staff were reluctant to go back to M-FiO2 after implementation of A-FiO2 as this reduced their workload (52). To our knowledge, there are no studies reporting parental experience with use of A-FiO2 either in clinical or research set-up.

In summary, currently there is good evidence to show that A-FiO2 is superior to M-FiO2 in maintaining SpO2 in TR and reducing extremes of SpO2 in preterm infants. However, there are no studies to support the clinical benefits of A-FiO2.

## What Is the Current Position of A-FiO2 in NICUs

Recent survey among UK neonatal units (192 units), showed that around 19 neonatal units (9.9%) units used A-FiO2 (54). Sixty-eight percent of the users used it in extreme preterm infants <26 weeks. Most responders to the survey reported higher ability to achieve proportion of time within the target SpO2 range and reduced need for manual adjustments. 89% of responders did not report any adverse outcomes. There were two reports that A-FiO2 resulted in inadvertent higher FiO2 when the probe was displaced and one report of masking event of desaturations.

The main challenges to implementation of A-FiO2 in NICU are lack of devices delivering A-FiO2, unfamiliarity with the devices and the lack of clinical outcome studies. Most of the new neonatal ventilators have A-FiO2 options on them. However, without appropriate expertise and training, the introduction and implementation of any change can be a failure. There are few reports that A-FiO2 can result in inadvertent higher FiO2 when the probe was displaced and mask desaturations.

## Potential and/or Perceived Barriers and Opportunities

### Masking of Clinical Deterioration

One of the concerns with regards to use of A-FiO2 is that it may mask clinical deteriorations. A-FiO2 is better than M-FiO2 at reducing hypoxemic episodes by automatically increasing the FiO2. However, the hypoxemic events may occur in relation to clinical deterioration like sepsis and just by increasing the FiO2 during these episodes, such events may be masked. This is generally not an issue especially if the staffing level is such that there is continuous close observation of these infants. This can also be overcome by appropriate staff training and using appropriate FiO2 alarms. In our unit we have addressed this by staff education and training. The CLiO2 system provides base FiO2 which is a trend, and a trend upwards may be indicative of deteriorating clinical condition. There is continuous scrutiny and medical staff are alerted when the there is an upwards trend of more than 5%.

### Hypoxemic Events Related to Apnea

Another potential limitation with A-FiO2 is its inability to differentiate hypoxemic events secondary to apneic episodes. A-FiO2 would provide sufficient oxygen to keep the SpO2 in TR, whereas with severe apneic episodes the infant may need other intervention like stimulation and positive pressure support. This issue can be overcome again by close observation of the infant and appropriate vital parameter alarm limits. Again, in these scenarios the role of staff education and training cannot be over emphasized.

### Average FiO2

It is often perceived at the bedside that FiO2 tends to be higher with A-FiO2 than M-FiO2. Some cross over studies with A-FiO2, did not show any statistical difference in the median FiO2 (32, 36, 42), was lower in A-FiO2 arm in Claure et al.'s study (31) and higher in Dijkman et al.'s study using PRICO (45).

### Lower SpO2 Median

Whilst on M-FiO2, the staff proactively intervene for hypoxia than hyperoxia episodes (11). Also, in a M-FiO2 set-up there is a tendency to keep the SpO2 in the upper range of the target (closer to 95%), whereas automated oxygen devices tend to target middle of SpO2 TR (close to 92–93%). This could potentially lead to lower mean/median SpO2 with A-FiO2. Whether this would have any impact on clinical outcomes needs to be studied and if needed this issue could be tackled with changes in algorithm. Further if such subtles of control are found to be warranted, shifting of A-FiO2 TR and alarm limits can be implemented.

### Disparity in SpO2 Readings Between the Monitors

In most of the A-FiO2 devices, the SpO2 can be monitored on the device in which the algorithm is incorporated. Some of the A-FiO2 devices albeit having the monitoring functionality does not have SpO2 alarms incorporated. This necessitates having additional SpO2 monitoring system with alarms to alert the staff of the deviation from TR. Despite using the same SpO2 technology, on occasions there seems to be discrepancy in SpO2 between the two monitoring devices. In our practice, we instruct our nursing staff to reposition/replace SpO2 probe which seems to resolve this discrepancy on most occasions. Resolution of discrepancy on most occasions reassures us that this discrepancy is not to the extent of clinical significance (hypoxia/hyperoxia), still it could result in staff and parental anxiety. However, this can be overcome by incorporating SpO2 monitoring with alarm limits on the same device.

### Cost-Effective and Staff Workload

Cost of the equipment is reported as another major limitation. Although, most of the newer neonatal ventilators are equipped with A-FiO2, the older versions may not have this facility. The discussion around cost-effectiveness should consider the clinical benefits with this technology. However, we are clearly lacking clinical studies looking at the short- and long-term outcomes of A-FiO2. When staff work load is considered, A-FiO2 has shown to be associated with significant reduction in the number of manual adjustments required thus allowing staff to focus on other aspects of clinical care (55).

### Customizing TR in Preterm Infants

Not all neonates of the exact same maturity are alike. The recent AAP guidelines recommends TR between 90 and 95%. However, it also underlines that there is no ideal TR and that it is patient specific and vary with gestation, chronological age and the underlying condition (56). Studies have shown that SGA are more susceptible to lower SpO2 (57). Also, the outcome data from individual centers may influence the TR used (58). A-FiO2 offers the potential to individualize TR according to the needs of the infant.

## Role of A-FiO2 in Neonatal Resuscitation

At birth, preterm infants slowly transition from fetal to neonatal life and often need interventions to support with this transition. Oxygen supplementation is often needed for these infants to maintain recommended SpO2 levels in the first 10 min of life. Hence use of pulse oximetry is recommended by the resuscitation council to monitor and titrate oxygen supplementation (59). With particular focus on reducing hyperoxemia and hypoxemia, most resuscitation councils recommended use of oxygen ranging from 21 to 30% for preterm infants at birth (59, 60).

Even with advances in neonatal resuscitation it remains a challenge to meet the SpO2 targets during the first 10 min of life. In a study with preterm infants ≤30 weeks the median percentages of time spent above and below the target were 44 and 51%, respectively (61). A-FiO2 could be one of the solutions to achieve the SpO2 targets at the time of birth. A study in ventilated preterm lambs showed a significant reduction in time spent above the SpO2 TR with the use of A-FiO2 using PRICO technology at birth (3). Use of A-FiO2 in resuscitation could be potentially useful and needs further research.

## Future Directions

- Need for RCTs that are adequately powered for short term and long-term outcomes. These studies should also report the cost effectiveness of the intervention, considering all the health outcomes and staff workload. The future studies should consider recruitment as soon as possible after birth to limit extremes of oxygenation during early period of the life.
- Studies are needed with characterization of all the existing algorithms with both invasive and non-invasive respiratory support.
- Innovations are needed to provide commercial algorithms that could support moving SpO2 targets (like during first 10 min of birth).
- Role of automated oxygen during elective neonatal intubation and reduction in hypoxemia during these procedures.
- Use of automated oxygen in preterm infants receiving nasal cannula low flow oxygen.
- Establish a role of A-FiO2 in low resource-staff limited settings.

## Conclusions

There is overwhelming evidence that A-FiO2 achieves higher proportion of time in SpO2 TR, reduces duration and episodes of hypoxemia and hyperoxemia. Although the impact on clinical outcomes associated with A-FiO2 is yet to be proven, from the available studies we can presume that there is no harm. Merely adopting the recommendations of targeting SpO2 (90–95%) will not suffice. It is essential that this is achieved. If not, this will be justice half done and infact we may not see the actual clinical benefits of SpO2 targeting. A-FiO2 is a promising technology that helps to achieve this target. However, the clinical benefits of it are still unknown.

## Author Contributions

VN, PL, and ML conceptualized the review, drafted the initial manuscript, reviewed, and revised the manuscript. TB reviewed and revised the manuscript. All authors approved the final manuscript as submitted and agree to be accountable for all aspects of the work.

## Conflict of Interest

The authors declare that the research was conducted in the absence of any commercial or financial relationships that could be construed as a potential conflict of interest.

## Publisher's Note

All claims expressed in this article are solely those of the authors and do not necessarily represent those of their affiliated organizations, or those of the publisher, the editors and the reviewers. Any product that may be evaluated in this article, or claim that may be made by its manufacturer, is not guaranteed or endorsed by the publisher.
